# Crystal structure of bis­[3-meth­oxy-17β-estra-1,3,5(10)-trien-17-yl] oxalate

**DOI:** 10.1107/S1600536814009349

**Published:** 2014-07-19

**Authors:** William T. A. Harrison, Lutfun Nahar, Alan B. Turner

**Affiliations:** a Department of Chemistry, University of Aberdeen, Meston Walk, Aberdeen AB24 3UE, Scotland

**Keywords:** crystal structure, oxalate, diester, steroid, conformation

## Abstract

In the title compound, C_40_H_50_O_6_, a symmetrical steroid oxalate diester, the dihedral angle between the CO_2_ planes of the oxalate linker is 61.5 (5)° and the C—C bond length is 1.513 (6) Å. The steroid *B*, *C* and *D* rings adopt half-chair, chair and envelope conformations, respectively, in both halves of the mol­ecule, which adopts an overall shallow V-shaped conformation. In the crystal, mol­ecules are linked by weak C—H⋯O inter­actions, forming a three-dimensional network.

## Abstract 2

The title symmetrical steroid oxalate diester is substantially twisted about the central O_2_C—CO_2_ bond, leading to an overall shallow V-shape for the molecule, which may correlate with its reactivity under flash vacuum pyrolysis. C—H⋯O hydrogen bonds help to establish the packing.

## Chemical context   

The pyrolysis of esters possessing aliphatic β-hydrogen atoms is a known route to alkenes *via* radical mediated β-elimination (Brown, 1980 ▶). As part of our studies in this area (Nahar, 2007 ▶), we now describe the crystal structure of the title compound, (I), an oxalate diester of 17-β-estradiol 3-methyl ether (Reck *et al.*, 1986 ▶; Schönnecker *et al.*, 2000 ▶). Flash-vacuum pyrolysis (FVP) of (I) at 873 K and 0.2 torr led to estra­tetra­ene 3-methyl ether in 47% yield.
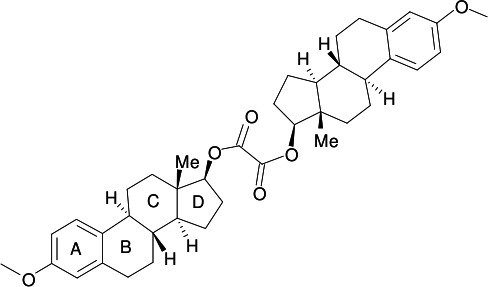


## Structural commentary   

**Figure 1:**
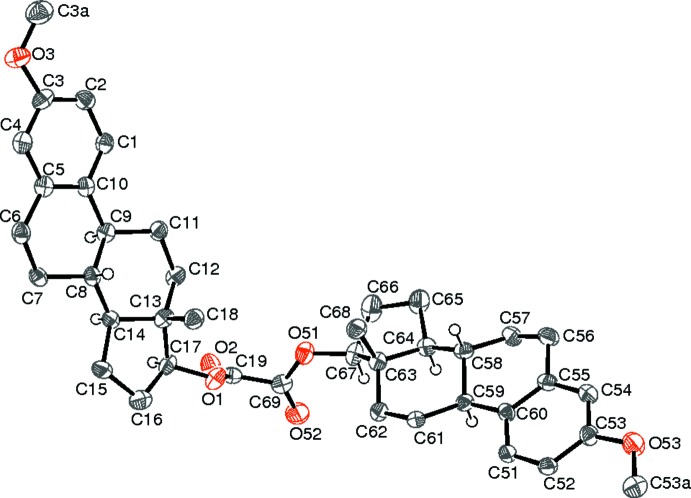
A view of the mol­ecular structure of the title mol­ecule, with atom labelling. Displacement ellipsoids are drawn at the 50% probability level. All the H atoms except those bonded to the chiral C atoms have been omitted for clarity.

The atom labelling scheme (Fig. 1 ▶) for (I) relates equivalent atoms in the two halves of the mol­ecule by adding 50, *e.g.* C1 and C51. The C19—C69 bond length of 1.513 (6) Å for the oxalate unit is exactly as expected for an *sp* ^2^–*sp* ^2^ carbon–carbon single bond but significantly shorter than the typical C—C bond length of about 1.57 Å in isolated oxalate ions (Dinnebier *et al.*, 2003 ▶). The mean C—O_C_ bond length is 1.324 Å and the mean C=O bond length is 1.197 Å. The dihedral angle between the C19/O1/O2 and C69/O51/O52 planes of 61.5 (5)° indicates a substantial twist. This leads to an overall shallow V-shaped conformation for the mol­ecule, with the C18 and C68 methyl groups facing each other [C18⋯C68 = 4.64 Å]. This could be significant in terms of the radical-reactivity of this mol­ecule under FVP (Nahar, 2007 ▶).

The meth­oxy carbon atom C3*A* is displaced from the C1–C5/C10 ring plane by −0.114 (7) Å. The C5–C10 ring conformation approximates to a half-chair with C7 and C8 displaced from the C5/C6/C9/C10 plane by 0.287 (7) and −0.477 (7) Å, respectively. The C8/C9/C11–C14 ring is a normal chair. The C13–C17 five-membered ring is an envelope, with C13 displaced from the mean plane of the other four C atoms by −0.735 (6) Å.

These ring conformations are essentially duplicated in the second half of the mol­ecule: C53*A* is displaced from the C51–C55/C60 plane by 0.096 (7) Å. For the C55–C60 ring, atoms C57 and C58 are displaced from the C55/C56/C59/C60 plane by −0.340 (7) and 0.422 (7) Å, respectively. The C58/C59/C61–C64 ring is a normal chair. The C63–C67 ring is an envelope, with C63 displaced from the mean plane of the other four atoms by 0.735 (6) Å.

The stereogenic centres in (I) have the following assumed chiralities: C8 *R*, C9 *S*, C13 *S*, C14 *S*, C17 *S*, C58 *R*, C59 *S*, C63 *S*, C64 *S*, C67 *S* to match the known absolute structure of the starting steroid (Reck *et al.*, 1986 ▶).

## Supra­molecular features   

In the crystal, mol­ecules are linked by weak C—H⋯O inter­actions (Table 1 ▶). Inter­estingly, these three bonds all arise from one ‘end’ of the mol­ecule. Two of these bonds are accepted by the same oxalate O atom and a three-dimensional network arises.

## Database survey   

In the closely related de­hydro­epiandrosterone oxalate diester (Cox *et al.*, 2007 ▶), the dihedral angles between the CO_2_ planes of the oxalate linkers in the two asymmetric mol­ecules are 24.2 (3) and 51.46 (11)°.

A search of the Cambridge Structural Database (Version 5.31**;** Allen & Motherwell, 2002 ▶) revealed four other structures containing an oxalate diester bridge between two fragments connected to the bridge by a secondary carbon atom. In C_22_H_34_O_4_ polymorph-I (Barnes & Weakley, 2004*a* ▶) the dihedral angle between the CO_2_ groups in the oxalate fragment is 12.5 (9)° and the bornyl substituents adopt a *syn* orientation. C_22_H_34_O_4_ polymorph-II (Barnes & Weakley, 2004*b* ▶) contains one-and-a-half mol­ecules in the asymmetric unit, with the half-mol­ecule completed by inversion symmetry, hence the oxalate bridge is planar by symmetry; in the complete mol­ecule, the oxalate dihedral angle is 12.2 (5)°. In both mol­ecules, the bornyl substituents are in an anti orientation.

In bis­(*cis*-(+)-2-(4-meth­oxy­phen­yl)-4-oxo-2,3,4,5-tetra­hydro-1,5-benzo­thia­zepin-3-yl) oxalate monohydrate (C_34_H_28_N_2_O_8_S_2_·H_2_O; Kumaradhas *et al.*, 2008 ▶), the oxalate dihedral angle is 27.2 (5)° with the substituents in an *anti* disposition. Finally, in bis­(di-*t*-butyl­meth­yl)oxalate (C_20_H_38_O_4_; Adiwidjaja & Voss, 1976 ▶), the oxalate unit is close to planar [dihedral angle = 5.6 (2)°], but the bulky substituents lie in a *syn* orientation.

## Synthesis and crystallization   

The title compound was prepared by the method of Lotowski & Guzmanski (2005 ▶) and recrystallized from di­chloro­methane/pyridine solution as colourless rods. M.p. 534–535 K; selected ^1^H NMR δ 0.86 (*s*, 18-Me), 3.74 (*s*, OMe), 4.79 (*m*, 17αH), 6.59 (*d*, 4-H), 6.67 (*dd*, 2-H), 7.16 (*d*, 1-H), ^13^C NMR δ 12.0, 23.3, 26.2, 27.2, 27.3, 29.7, 36.8, 38.5, 43.3, 43.7, 49.7, 55.2, 85.3, 111.5, 113.8, 126.3,132.3, 137,8, 157.5, 158.2.

## Refinement   

The crystal quality was only fair, which may correlate with the rather high *R*_int_ value. The H atoms were placed in calculated positions (C—H = 0.95–0.99 Å) and refined as riding atoms with *U*_iso_(H) = 1.2*U*_eq_(C) or 1.5*U*_eq_(methyl C). The methyl groups were allowed to rotate, but not to tip, to best fit the electron density. Experimental details are given in Table 2 ▶.

## Supplementary Material

Crystal structure: contains datablock(s) I, global. DOI: 10.1107/S1600536814009349/su0001sup1.cif

Structure factors: contains datablock(s) I. DOI: 10.1107/S1600536814009349/su0001Isup2.hkl

CCDC reference: 1004276

Additional supporting information:  crystallographic information; 3D view; checkCIF report

## Figures and Tables

**Figure 2:**
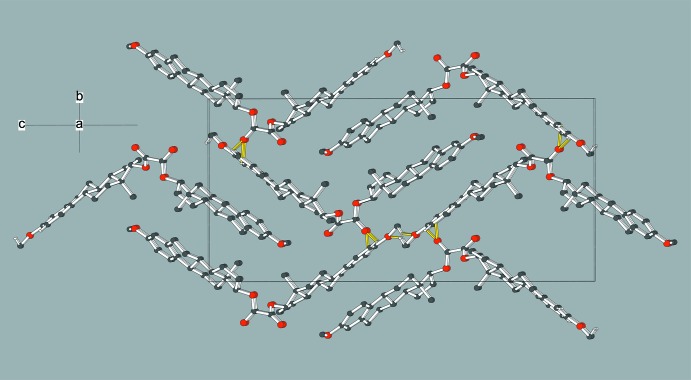
The packing in (I) viewed down [100] with C—H⋯O hydrogen bonds indicated by yellow lines. All H atoms not involved in such inter­actions have been omitted for clarity.

**Table 1:** Hydrogen-bond geometry (Å, °)

*D*—H⋯*A*	*D*—H	H⋯*A*	*D*⋯*A*	*D*—H⋯*A*
C52—H52⋯O2^i^	0.95	2.51	3.161 (5)	126
C53*A*—H53*B*⋯O53^ii^	0.98	2.51	3.378 (6)	147
C54—H54⋯O2^iii^	0.95	2.56	3.309 (5)	136

Symmetry codes: (i) 
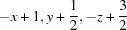
; (ii) 
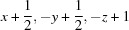
; (iii) 
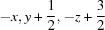
.

**Table 2:** Experimental details

Crystal data	
Chemical formula	C_40_H_50_O_6_
*M*_r_	626.80
Crystal system, space group	Orthorhombic, *P*2_1_2_1_2_1_
Temperature (K)	120
*a*, *b*, *c* (Å)	7.8559 (4), 14.1579 (10), 29.888 (2)
*V* (Å^3^)	3324.2 (4)
*Z*	4
Radiation type	Mo *K*α
μ (mm^−1^)	0.08
Crystal size (mm)	0.25 × 0.08 × 0.06

Data collection	
Diffractometer	Nonius KappaCCD
No. of measured, independent and observed [*I* > 2σ(*I*)] reflections	21809, 3674, 2220
*R*_int_	0.169
(sin θ/λ)_max_ (Å^−1^)	0.617

Refinement	
*R*[*F* ^2^ > 2σ(*F* ^2^)], *wR*(*F* ^2^), *S*	0.075, 0.128, 1.02
No. of reflections	3674
No. of parameters	420
H-atom treatment	H-atom parameters constrained
Δρ_max_, Δρ_min_ (e Å^−3^)	0.32, −0.31

Computer programs: *COLLECT* (Nonius, 1998 ▶), *DENZO* and *SCALEPACK* (Otwinowski & Minor, 1997 ▶), *SORTAV* (Blessing, 1995 ▶), *SHELXS97* and *SHELXL97* (Sheldrick, 2008 ▶) and *ORTEP-3 for Windows* (Farrugia, 2012 ▶).

## supplementary crystallographic information

### Crystal data

**Table:**

C_40_H_50_O_6_	*F*(000) = 1352
*M**_r_* = 626.80	*D*_x_ = 1.252 Mg m^−^^3^
Orthorhombic, *P*2_1_2_1_2_1_	Mo *K*α radiation, λ = 0.71073 Å
Hall symbol: P 2ac 2ab	Cell parameters from 4069 reflections
*a* = 7.8559 (4) Å	θ = 1.0–27.5°
*b* = 14.1579 (10) Å	µ = 0.08 mm^−^^1^
*c* = 29.888 (2) Å	*T* = 120 K
*V* = 3324.2 (4) Å^3^	Rod, colourless
*Z* = 4	0.25 × 0.08 × 0.06 mm

### Data collection

**Table:**

Nonius KappaCCD diffractometer	2220 reflections with *I* > 2σ(*I*)
Radiation source: fine-focus sealed tube	*R*_int_ = 0.169
Graphite monochromator	θ_max_ = 26.0°, θ_min_ = 2.9°
ω and φ scans	*h* = −9→8
21809 measured reflections	*k* = −17→17
3674 independent reflections	*l* = −35→36

### Refinement

**Table:**

Refinement on *F*^2^	Secondary atom site location: difference Fourier map
Least-squares matrix: full	Hydrogen site location: inferred from neighbouring sites
*R*[*F*^2^ > 2σ(*F*^2^)] = 0.075	H-atom parameters constrained
*wR*(*F*^2^) = 0.128	*w* = 1/[σ^2^(*F*_o_^2^) + (0.0633*P*)^2^] where *P* = (*F*_o_^2^ + 2*F*_c_^2^)/3
*S* = 1.02	(Δ/σ)_max_ < 0.001
3674 reflections	Δρ_max_ = 0.32 e Å^−^^3^
420 parameters	Δρ_min_ = −0.31 e Å^−^^3^
0 restraints	Extinction correction: *SHELXL97* (Sheldrick, 2008), Fc^*^=kFc[1+0.001xFc^2^λ^3^/sin(2θ)]^-1/4^
Primary atom site location: structure-invariant direct methods	Extinction coefficient: 0.0132 (14)

### Special details

**Table:**

Geometry. All e.s.d.'s (except the e.s.d. in the dihedral angle between two l.s. planes) are estimated using the full covariance matrix. The cell e.s.d.'s are taken into account individually in the estimation of e.s.d.'s in distances, angles and torsion angles; correlations between e.s.d.'s in cell parameters are only used when they are defined by crystal symmetry. An approximate (isotropic) treatment of cell e.s.d.'s is used for estimating e.s.d.'s involving l.s. planes.
Refinement. Refinement of *F*^2^ against ALL reflections. The weighted *R*-factor *wR* and goodness of fit *S* are based on *F*^2^, conventional *R*-factors *R* are based on *F*, with *F* set to zero for negative *F*^2^. The threshold expression of *F*^2^ > σ(*F*^2^) is used only for calculating *R*-factors(gt) *etc*. and is not relevant to the choice of reflections for refinement. *R*-factors based on *F*^2^ are statistically about twice as large as those based on *F*, and *R*- factors based on ALL data will be even larger.

### Fractional atomic coordinates and isotropic or equivalent isotropic displacement parameters (Å^2^)

**Table:**

	*x*	*y*	*z*	*U*_iso_*/*U*_eq_	
C1	0.1936 (5)	0.1231 (3)	1.09702 (12)	0.0288 (10)	
H1	0.1244	0.0772	1.0826	0.035*	
C2	0.1253 (6)	0.1738 (3)	1.13226 (13)	0.0320 (11)	
H2	0.0108	0.1639	1.1413	0.038*	
C3	0.2256 (6)	0.2390 (3)	1.15401 (13)	0.0317 (11)	
C3A	0.0043 (6)	0.2884 (3)	1.20355 (15)	0.0525 (14)	
H3A	−0.0151	0.3322	1.2285	0.079*	
H3B	−0.0228	0.2240	1.2131	0.079*	
H3C	−0.0687	0.3060	1.1783	0.079*	
C4	0.3910 (6)	0.2540 (3)	1.13966 (13)	0.0308 (11)	
H4	0.4600	0.2991	1.1547	0.037*	
C5	0.4578 (5)	0.2042 (3)	1.10366 (12)	0.0283 (10)	
C6	0.6359 (5)	0.2281 (3)	1.08821 (13)	0.0316 (11)	
H6A	0.7095	0.2364	1.1148	0.038*	
H6B	0.6328	0.2891	1.0720	0.038*	
C7	0.7154 (5)	0.1538 (3)	1.05784 (12)	0.0284 (10)	
H7A	0.8183	0.1802	1.0434	0.034*	
H7B	0.7503	0.0985	1.0759	0.034*	
C8	0.5891 (5)	0.1231 (3)	1.02223 (12)	0.0268 (10)	
H8	0.5479	0.1803	1.0059	0.032*	
C9	0.4351 (5)	0.0748 (3)	1.04514 (12)	0.0275 (10)	
H9	0.4824	0.0180	1.0607	0.033*	
C10	0.3587 (5)	0.1362 (3)	1.08178 (13)	0.0259 (10)	
C11	0.3043 (5)	0.0365 (3)	1.01134 (13)	0.0323 (10)	
H11A	0.2483	0.0902	0.9961	0.039*	
H11B	0.2157	0.0006	1.0276	0.039*	
C12	0.3877 (5)	−0.0279 (3)	0.97611 (13)	0.0315 (11)	
H12A	0.4284	−0.0865	0.9908	0.038*	
H12B	0.3019	−0.0458	0.9534	0.038*	
C13	0.5359 (6)	0.0206 (3)	0.95328 (12)	0.0276 (10)	
C14	0.6641 (5)	0.0544 (3)	0.98867 (12)	0.0253 (10)	
H14	0.6981	−0.0029	1.0061	0.030*	
C15	0.8217 (5)	0.0831 (3)	0.96073 (13)	0.0347 (11)	
H15A	0.9277	0.0744	0.9782	0.042*	
H15B	0.8138	0.1499	0.9512	0.042*	
C16	0.8178 (6)	0.0159 (3)	0.91974 (14)	0.0381 (12)	
H16A	0.8119	0.0521	0.8915	0.046*	
H16B	0.9200	−0.0250	0.9191	0.046*	
C17	0.6567 (6)	−0.0427 (3)	0.92658 (12)	0.0313 (11)	
H17	0.6848	−0.1001	0.9447	0.038*	
C18	0.4706 (6)	0.1014 (3)	0.92326 (13)	0.0335 (11)	
H18A	0.5671	0.1314	0.9081	0.050*	
H18B	0.4114	0.1483	0.9417	0.050*	
H18C	0.3918	0.0758	0.9009	0.050*	
C51	0.1276 (5)	0.1300 (3)	0.61705 (12)	0.0272 (10)	
H51	0.2452	0.1250	0.6241	0.033*	
C52	0.0804 (5)	0.1861 (3)	0.58125 (13)	0.0276 (10)	
H52	0.1635	0.2193	0.5643	0.033*	
C53	−0.0910 (6)	0.1929 (3)	0.57065 (13)	0.0292 (10)	
C53A	−0.0362 (6)	0.3019 (3)	0.51109 (13)	0.0377 (11)	
H53A	−0.0958	0.3338	0.4866	0.057*	
H53B	0.0542	0.2618	0.4988	0.057*	
H53C	0.0137	0.3491	0.5311	0.057*	
C54	−0.2088 (6)	0.1447 (3)	0.59550 (13)	0.0323 (10)	
H54	−0.3258	0.1494	0.5878	0.039*	
C55	−0.1614 (5)	0.0891 (3)	0.63183 (13)	0.0283 (10)	
C56	−0.2988 (5)	0.0420 (3)	0.65920 (14)	0.0340 (11)	
H56A	−0.3869	0.0169	0.6387	0.041*	
H56B	−0.3536	0.0900	0.6785	0.041*	
C57	−0.2337 (5)	−0.0382 (3)	0.68852 (14)	0.0332 (11)	
H57A	−0.3233	−0.0571	0.7101	0.040*	
H57B	−0.2064	−0.0937	0.6696	0.040*	
C58	−0.0757 (5)	−0.0071 (3)	0.71376 (13)	0.0287 (10)	
H58	−0.1030	0.0519	0.7307	0.034*	
C59	0.0704 (5)	0.0145 (3)	0.68065 (12)	0.0269 (10)	
H59	0.0982	−0.0469	0.6658	0.032*	
C60	0.0123 (5)	0.0806 (3)	0.64322 (12)	0.0248 (10)	
C61	0.2325 (5)	0.0455 (3)	0.70446 (12)	0.0304 (10)	
H61A	0.2123	0.1068	0.7195	0.037*	
H61B	0.3241	0.0546	0.6821	0.037*	
C62	0.2897 (6)	−0.0270 (3)	0.73903 (13)	0.0328 (10)	
H62A	0.3248	−0.0856	0.7235	0.039*	
H62B	0.3898	−0.0022	0.7553	0.039*	
C63	0.1492 (5)	−0.0500 (3)	0.77237 (12)	0.0286 (10)	
C64	−0.0115 (5)	−0.0810 (3)	0.74658 (13)	0.0303 (10)	
H64	0.0239	−0.1363	0.7280	0.036*	
C65	−0.1299 (6)	−0.1210 (3)	0.78286 (13)	0.0441 (13)	
H65A	−0.2075	−0.1694	0.7703	0.053*	
H65B	−0.1984	−0.0703	0.7969	0.053*	
C66	−0.0042 (6)	−0.1654 (3)	0.81707 (16)	0.0505 (14)	
H66A	−0.0224	−0.1386	0.8473	0.061*	
H66B	−0.0191	−0.2347	0.8185	0.061*	
C67	0.1733 (6)	−0.1400 (3)	0.79949 (13)	0.0376 (12)	
H67	0.2157	−0.1917	0.7795	0.045*	
C68	0.1171 (6)	0.0345 (3)	0.80322 (13)	0.0346 (11)	
H68A	0.0937	0.0908	0.7851	0.052*	
H68B	0.2180	0.0457	0.8218	0.052*	
H68C	0.0191	0.0212	0.8225	0.052*	
C19	0.5300 (5)	−0.1607 (3)	0.88064 (14)	0.0311 (11)	
C69	0.4324 (6)	−0.1782 (3)	0.83780 (14)	0.0327 (11)	
O1	0.5808 (4)	−0.07180 (19)	0.88412 (8)	0.0351 (8)	
O2	0.5552 (4)	−0.2225 (2)	0.90715 (10)	0.0454 (9)	
O3	0.1760 (4)	0.2930 (2)	1.19035 (9)	0.0447 (9)	
O51	0.2916 (4)	−0.12707 (19)	0.83696 (9)	0.0375 (8)	
O52	0.4778 (4)	−0.2347 (2)	0.81047 (10)	0.0546 (10)	
O53	−0.1536 (4)	0.2451 (2)	0.53540 (9)	0.0406 (8)	

### Atomic displacement parameters (Å^2^)

**Table:**

	*U*^11^	*U*^22^	*U*^33^	*U*^12^	*U*^13^	*U*^23^
C1	0.032 (3)	0.026 (2)	0.028 (2)	−0.003 (2)	−0.003 (2)	0.0007 (18)
C2	0.030 (3)	0.035 (2)	0.031 (2)	0.000 (2)	0.002 (2)	0.005 (2)
C3	0.046 (3)	0.026 (2)	0.023 (2)	0.009 (2)	0.001 (2)	0.0015 (18)
C3A	0.061 (4)	0.051 (3)	0.046 (3)	0.017 (3)	0.004 (3)	−0.003 (2)
C4	0.037 (3)	0.023 (2)	0.032 (2)	−0.002 (2)	−0.004 (2)	0.0001 (19)
C5	0.034 (3)	0.022 (2)	0.029 (2)	−0.001 (2)	−0.003 (2)	0.0044 (18)
C6	0.032 (3)	0.031 (3)	0.032 (2)	−0.008 (2)	−0.004 (2)	0.0015 (19)
C7	0.024 (3)	0.028 (2)	0.033 (2)	−0.004 (2)	−0.001 (2)	0.0029 (18)
C8	0.026 (3)	0.027 (2)	0.027 (2)	−0.004 (2)	−0.0035 (19)	0.0018 (18)
C9	0.033 (3)	0.023 (2)	0.027 (2)	−0.003 (2)	−0.001 (2)	0.0030 (17)
C10	0.029 (3)	0.022 (2)	0.026 (2)	−0.002 (2)	−0.003 (2)	0.0055 (18)
C11	0.031 (3)	0.037 (2)	0.028 (2)	−0.011 (2)	0.000 (2)	−0.0013 (19)
C12	0.039 (3)	0.027 (2)	0.028 (2)	−0.008 (2)	0.003 (2)	−0.0022 (18)
C13	0.037 (3)	0.022 (2)	0.025 (2)	−0.002 (2)	0.002 (2)	0.0045 (17)
C14	0.024 (3)	0.027 (2)	0.025 (2)	0.0007 (19)	−0.0010 (19)	0.0077 (17)
C15	0.025 (3)	0.041 (3)	0.037 (2)	−0.005 (2)	0.005 (2)	−0.003 (2)
C16	0.040 (3)	0.040 (3)	0.035 (2)	0.005 (2)	0.006 (2)	0.003 (2)
C17	0.042 (3)	0.032 (2)	0.020 (2)	0.006 (2)	−0.003 (2)	0.0003 (18)
C18	0.037 (3)	0.034 (3)	0.029 (2)	0.005 (2)	−0.001 (2)	0.0014 (19)
C51	0.023 (2)	0.032 (2)	0.026 (2)	0.001 (2)	−0.001 (2)	−0.0046 (19)
C52	0.025 (3)	0.032 (2)	0.026 (2)	−0.001 (2)	0.003 (2)	−0.0046 (19)
C53	0.030 (3)	0.029 (2)	0.029 (2)	0.005 (2)	−0.003 (2)	0.0032 (19)
C53A	0.041 (3)	0.036 (3)	0.035 (2)	0.001 (2)	0.004 (2)	0.001 (2)
C54	0.024 (3)	0.040 (3)	0.033 (2)	0.001 (2)	0.000 (2)	0.002 (2)
C55	0.025 (3)	0.029 (2)	0.031 (2)	0.002 (2)	0.004 (2)	−0.0032 (18)
C56	0.026 (3)	0.038 (3)	0.038 (2)	0.004 (2)	0.003 (2)	−0.004 (2)
C57	0.026 (3)	0.037 (3)	0.037 (2)	−0.006 (2)	0.000 (2)	0.000 (2)
C58	0.028 (3)	0.028 (2)	0.030 (2)	−0.001 (2)	0.005 (2)	−0.0043 (18)
C59	0.028 (3)	0.029 (2)	0.024 (2)	−0.001 (2)	0.0016 (19)	−0.0042 (17)
C60	0.022 (3)	0.024 (2)	0.028 (2)	0.000 (2)	−0.003 (2)	−0.0050 (18)
C61	0.025 (3)	0.043 (3)	0.024 (2)	−0.001 (2)	0.003 (2)	0.0011 (19)
C62	0.031 (3)	0.040 (3)	0.027 (2)	0.004 (2)	−0.002 (2)	−0.0039 (19)
C63	0.031 (3)	0.026 (2)	0.028 (2)	0.001 (2)	−0.001 (2)	−0.0004 (18)
C64	0.031 (3)	0.029 (2)	0.031 (2)	−0.001 (2)	0.001 (2)	−0.0008 (18)
C65	0.047 (3)	0.048 (3)	0.037 (3)	−0.015 (2)	−0.003 (2)	0.013 (2)
C66	0.057 (4)	0.049 (3)	0.046 (3)	−0.021 (3)	−0.011 (3)	0.012 (2)
C67	0.046 (3)	0.037 (3)	0.030 (2)	0.000 (2)	−0.009 (2)	−0.001 (2)
C68	0.035 (3)	0.035 (3)	0.033 (2)	−0.001 (2)	0.001 (2)	−0.0058 (19)
C19	0.028 (3)	0.031 (3)	0.034 (2)	0.004 (2)	0.006 (2)	−0.001 (2)
C69	0.039 (3)	0.026 (2)	0.034 (2)	0.000 (2)	0.005 (2)	0.002 (2)
O1	0.052 (2)	0.0255 (16)	0.0274 (15)	−0.0023 (15)	−0.0004 (15)	−0.0002 (12)
O2	0.045 (2)	0.0339 (19)	0.058 (2)	−0.0012 (16)	−0.0153 (18)	0.0103 (16)
O3	0.047 (2)	0.046 (2)	0.0405 (18)	0.0055 (17)	0.0089 (17)	−0.0084 (15)
O51	0.046 (2)	0.0330 (17)	0.0334 (16)	0.0086 (17)	−0.0078 (16)	−0.0033 (14)
O52	0.048 (2)	0.062 (2)	0.054 (2)	0.0107 (19)	0.0021 (18)	−0.0253 (18)
O53	0.0330 (19)	0.0466 (19)	0.0421 (17)	−0.0020 (15)	−0.0014 (16)	0.0135 (15)

### Geometric parameters (Å, º)

**Table:**

C1—C2	1.383 (5)	C52—C53	1.387 (6)
C1—C10	1.387 (5)	C52—H52	0.9500
C1—H1	0.9500	C53—C54	1.369 (5)
C2—C3	1.376 (6)	C53—O53	1.378 (4)
C2—H2	0.9500	C53A—O53	1.423 (5)
C3—O3	1.384 (5)	C53A—H53A	0.9800
C3—C4	1.385 (6)	C53A—H53B	0.9800
C3A—O3	1.407 (5)	C53A—H53C	0.9800
C3A—H3A	0.9800	C54—C55	1.392 (5)
C3A—H3B	0.9800	C54—H54	0.9500
C3A—H3C	0.9800	C55—C60	1.412 (5)
C4—C5	1.390 (5)	C55—C56	1.509 (6)
C4—H4	0.9500	C56—C57	1.523 (5)
C5—C10	1.400 (5)	C56—H56A	0.9900
C5—C6	1.512 (6)	C56—H56B	0.9900
C6—C7	1.523 (5)	C57—C58	1.518 (5)
C6—H6A	0.9900	C57—H57A	0.9900
C6—H6B	0.9900	C57—H57B	0.9900
C7—C8	1.519 (5)	C58—C64	1.521 (5)
C7—H7A	0.9900	C58—C59	1.546 (6)
C7—H7B	0.9900	C58—H58	1.0000
C8—C14	1.516 (5)	C59—C61	1.524 (5)
C8—C9	1.549 (5)	C59—C60	1.528 (5)
C8—H8	1.0000	C59—H59	1.0000
C9—C10	1.523 (5)	C61—C62	1.524 (5)
C9—C11	1.539 (5)	C61—H61A	0.9900
C9—H9	1.0000	C61—H61B	0.9900
C11—C12	1.540 (5)	C62—C63	1.522 (5)
C11—H11A	0.9900	C62—H62A	0.9900
C11—H11B	0.9900	C62—H62B	0.9900
C12—C13	1.514 (5)	C63—C67	1.523 (5)
C12—H12A	0.9900	C63—C68	1.531 (5)
C12—H12B	0.9900	C63—C64	1.544 (6)
C13—C17	1.530 (5)	C64—C65	1.537 (6)
C13—C14	1.537 (5)	C64—H64	1.0000
C13—C18	1.542 (5)	C65—C66	1.554 (6)
C14—C15	1.547 (5)	C65—H65A	0.9900
C14—H14	1.0000	C65—H65B	0.9900
C15—C16	1.552 (5)	C66—C67	1.533 (6)
C15—H15A	0.9900	C66—H66A	0.9900
C15—H15B	0.9900	C66—H66B	0.9900
C16—C17	1.527 (6)	C67—O51	1.467 (5)
C16—H16A	0.9900	C67—H67	1.0000
C16—H16B	0.9900	C68—H68A	0.9800
C17—O1	1.461 (4)	C68—H68B	0.9800
C17—H17	1.0000	C68—H68C	0.9800
C18—H18A	0.9800	C19—O2	1.197 (5)
C18—H18B	0.9800	C19—O1	1.325 (5)
C18—H18C	0.9800	C19—C69	1.513 (6)
C51—C52	1.383 (5)	C69—O52	1.198 (5)
C51—C60	1.386 (5)	C69—O51	1.322 (5)
C51—H51	0.9500		
			
C2—C1—C10	122.9 (4)	C53—C52—H52	120.8
C2—C1—H1	118.6	C54—C53—O53	116.2 (4)
C10—C1—H1	118.6	C54—C53—C52	119.9 (4)
C3—C2—C1	119.1 (4)	O53—C53—C52	123.9 (4)
C3—C2—H2	120.5	O53—C53A—H53A	109.5
C1—C2—H2	120.5	O53—C53A—H53B	109.5
C2—C3—O3	125.5 (4)	H53A—C53A—H53B	109.5
C2—C3—C4	119.6 (4)	O53—C53A—H53C	109.5
O3—C3—C4	115.0 (4)	H53A—C53A—H53C	109.5
O3—C3A—H3A	109.5	H53B—C53A—H53C	109.5
O3—C3A—H3B	109.5	C53—C54—C55	121.6 (4)
H3A—C3A—H3B	109.5	C53—C54—H54	119.2
O3—C3A—H3C	109.5	C55—C54—H54	119.2
H3A—C3A—H3C	109.5	C54—C55—C60	119.6 (4)
H3B—C3A—H3C	109.5	C54—C55—C56	118.8 (4)
C3—C4—C5	121.1 (4)	C60—C55—C56	121.5 (4)
C3—C4—H4	119.5	C55—C56—C57	113.6 (3)
C5—C4—H4	119.5	C55—C56—H56A	108.8
C4—C5—C10	120.1 (4)	C57—C56—H56A	108.8
C4—C5—C6	118.2 (4)	C55—C56—H56B	108.8
C10—C5—C6	121.7 (4)	C57—C56—H56B	108.8
C5—C6—C7	114.0 (3)	H56A—C56—H56B	107.7
C5—C6—H6A	108.7	C58—C57—C56	110.1 (3)
C7—C6—H6A	108.7	C58—C57—H57A	109.6
C5—C6—H6B	108.7	C56—C57—H57A	109.6
C7—C6—H6B	108.7	C58—C57—H57B	109.6
H6A—C6—H6B	107.6	C56—C57—H57B	109.6
C8—C7—C6	110.3 (3)	H57A—C57—H57B	108.2
C8—C7—H7A	109.6	C57—C58—C64	113.0 (3)
C6—C7—H7A	109.6	C57—C58—C59	110.3 (3)
C8—C7—H7B	109.6	C64—C58—C59	107.6 (3)
C6—C7—H7B	109.6	C57—C58—H58	108.6
H7A—C7—H7B	108.1	C64—C58—H58	108.6
C14—C8—C7	113.2 (3)	C59—C58—H58	108.6
C14—C8—C9	108.2 (3)	C61—C59—C60	114.5 (3)
C7—C8—C9	109.1 (3)	C61—C59—C58	112.2 (3)
C14—C8—H8	108.8	C60—C59—C58	111.6 (3)
C7—C8—H8	108.8	C61—C59—H59	105.9
C9—C8—H8	108.8	C60—C59—H59	105.9
C10—C9—C11	114.2 (3)	C58—C59—H59	105.9
C10—C9—C8	111.9 (3)	C51—C60—C55	116.9 (4)
C11—C9—C8	112.7 (3)	C51—C60—C59	121.8 (4)
C10—C9—H9	105.7	C55—C60—C59	121.1 (4)
C11—C9—H9	105.7	C59—C61—C62	111.7 (3)
C8—C9—H9	105.7	C59—C61—H61A	109.3
C1—C10—C5	117.3 (4)	C62—C61—H61A	109.3
C1—C10—C9	121.9 (4)	C59—C61—H61B	109.3
C5—C10—C9	120.6 (4)	C62—C61—H61B	109.3
C9—C11—C12	111.9 (3)	H61A—C61—H61B	107.9
C9—C11—H11A	109.2	C63—C62—C61	111.9 (3)
C12—C11—H11A	109.2	C63—C62—H62A	109.2
C9—C11—H11B	109.2	C61—C62—H62A	109.2
C12—C11—H11B	109.2	C63—C62—H62B	109.2
H11A—C11—H11B	107.9	C61—C62—H62B	109.2
C13—C12—C11	111.5 (3)	H62A—C62—H62B	107.9
C13—C12—H12A	109.3	C62—C63—C67	115.9 (3)
C11—C12—H12A	109.3	C62—C63—C68	110.3 (3)
C13—C12—H12B	109.3	C67—C63—C68	110.8 (3)
C11—C12—H12B	109.3	C62—C63—C64	109.1 (3)
H12A—C12—H12B	108.0	C67—C63—C64	97.4 (3)
C12—C13—C17	116.5 (3)	C68—C63—C64	112.9 (3)
C12—C13—C14	109.6 (3)	C58—C64—C65	120.5 (4)
C17—C13—C14	97.8 (3)	C58—C64—C63	113.4 (3)
C12—C13—C18	110.1 (4)	C65—C64—C63	104.3 (3)
C17—C13—C18	109.7 (3)	C58—C64—H64	105.8
C14—C13—C18	112.8 (3)	C65—C64—H64	105.8
C8—C14—C13	113.6 (3)	C63—C64—H64	105.8
C8—C14—C15	120.0 (3)	C64—C65—C66	103.2 (4)
C13—C14—C15	103.6 (3)	C64—C65—H65A	111.1
C8—C14—H14	106.2	C66—C65—H65A	111.1
C13—C14—H14	106.2	C64—C65—H65B	111.1
C15—C14—H14	106.2	C66—C65—H65B	111.1
C14—C15—C16	104.4 (3)	H65A—C65—H65B	109.1
C14—C15—H15A	110.9	C67—C66—C65	105.0 (3)
C16—C15—H15A	110.9	C67—C66—H66A	110.8
C14—C15—H15B	110.9	C65—C66—H66A	110.8
C16—C15—H15B	110.9	C67—C66—H66B	110.8
H15A—C15—H15B	108.9	C65—C66—H66B	110.8
C17—C16—C15	104.1 (3)	H66A—C66—H66B	108.8
C17—C16—H16A	110.9	O51—C67—C63	112.4 (3)
C15—C16—H16A	110.9	O51—C67—C66	110.1 (3)
C17—C16—H16B	110.9	C63—C67—C66	105.4 (4)
C15—C16—H16B	110.9	O51—C67—H67	109.6
H16A—C16—H16B	109.0	C63—C67—H67	109.6
O1—C17—C16	112.0 (3)	C66—C67—H67	109.6
O1—C17—C13	111.4 (3)	C63—C68—H68A	109.5
C16—C17—C13	105.4 (3)	C63—C68—H68B	109.5
O1—C17—H17	109.3	H68A—C68—H68B	109.5
C16—C17—H17	109.3	C63—C68—H68C	109.5
C13—C17—H17	109.3	H68A—C68—H68C	109.5
C13—C18—H18A	109.5	H68B—C68—H68C	109.5
C13—C18—H18B	109.5	O2—C19—O1	126.4 (4)
H18A—C18—H18B	109.5	O2—C19—C69	121.7 (4)
C13—C18—H18C	109.5	O1—C19—C69	112.0 (3)
H18A—C18—H18C	109.5	O52—C69—O51	127.0 (4)
H18B—C18—H18C	109.5	O52—C69—C19	122.4 (4)
C52—C51—C60	123.4 (4)	O51—C69—C19	110.5 (4)
C52—C51—H51	118.3	C19—O1—C17	117.3 (3)
C60—C51—H51	118.3	C3—O3—C3A	117.7 (4)
C51—C52—C53	118.5 (4)	C69—O51—C67	118.4 (3)
C51—C52—H52	120.8	C53—O53—C53A	117.5 (3)
			
C10—C1—C2—C3	−1.5 (6)	C60—C55—C56—C57	−19.4 (5)
C1—C2—C3—O3	−178.3 (3)	C55—C56—C57—C58	47.5 (4)
C1—C2—C3—C4	1.4 (6)	C56—C57—C58—C64	175.4 (3)
C2—C3—C4—C5	−0.1 (6)	C56—C57—C58—C59	−64.0 (4)
O3—C3—C4—C5	179.7 (3)	C57—C58—C59—C61	−179.6 (3)
C3—C4—C5—C10	−1.2 (6)	C64—C58—C59—C61	−55.9 (4)
C3—C4—C5—C6	176.7 (4)	C57—C58—C59—C60	50.3 (4)
C4—C5—C6—C7	164.2 (3)	C64—C58—C59—C60	174.0 (3)
C10—C5—C6—C7	−18.0 (5)	C52—C51—C60—C55	0.3 (6)
C5—C6—C7—C8	45.8 (4)	C52—C51—C60—C59	176.1 (3)
C6—C7—C8—C14	175.7 (3)	C54—C55—C60—C51	0.4 (6)
C6—C7—C8—C9	−63.7 (4)	C56—C55—C60—C51	−177.3 (4)
C14—C8—C9—C10	176.1 (3)	C54—C55—C60—C59	−175.4 (3)
C7—C8—C9—C10	52.6 (4)	C56—C55—C60—C59	6.9 (6)
C14—C8—C9—C11	−53.5 (4)	C61—C59—C60—C51	33.3 (5)
C7—C8—C9—C11	−177.0 (3)	C58—C59—C60—C51	162.2 (3)
C2—C1—C10—C5	0.1 (6)	C61—C59—C60—C55	−151.1 (4)
C2—C1—C10—C9	175.6 (4)	C58—C59—C60—C55	−22.2 (5)
C4—C5—C10—C1	1.2 (5)	C60—C59—C61—C62	−176.0 (3)
C6—C5—C10—C1	−176.6 (4)	C58—C59—C61—C62	55.5 (4)
C4—C5—C10—C9	−174.3 (3)	C59—C61—C62—C63	−54.6 (4)
C6—C5—C10—C9	7.9 (5)	C61—C62—C63—C67	163.1 (3)
C11—C9—C10—C1	29.8 (5)	C61—C62—C63—C68	−70.1 (4)
C8—C9—C10—C1	159.4 (3)	C61—C62—C63—C64	54.4 (4)
C11—C9—C10—C5	−154.9 (3)	C57—C58—C64—C65	−55.4 (5)
C8—C9—C10—C5	−25.3 (5)	C59—C58—C64—C65	−177.4 (3)
C10—C9—C11—C12	−178.0 (3)	C57—C58—C64—C63	180.0 (3)
C8—C9—C11—C12	52.8 (4)	C59—C58—C64—C63	57.9 (4)
C9—C11—C12—C13	−53.5 (4)	C62—C63—C64—C58	−58.0 (4)
C11—C12—C13—C17	165.0 (3)	C67—C63—C64—C58	−178.8 (3)
C11—C12—C13—C14	55.3 (4)	C68—C63—C64—C58	64.9 (4)
C11—C12—C13—C18	−69.3 (4)	C62—C63—C64—C65	168.9 (3)
C7—C8—C14—C13	178.3 (3)	C67—C63—C64—C65	48.2 (4)
C9—C8—C14—C13	57.3 (4)	C68—C63—C64—C65	−68.1 (4)
C7—C8—C14—C15	−58.4 (5)	C58—C64—C65—C66	−161.9 (4)
C9—C8—C14—C15	−179.5 (3)	C63—C64—C65—C66	−33.1 (4)
C12—C13—C14—C8	−59.3 (4)	C64—C65—C66—C67	4.5 (4)
C17—C13—C14—C8	178.9 (3)	C62—C63—C67—O51	79.5 (4)
C18—C13—C14—C8	63.7 (4)	C68—C63—C67—O51	−47.2 (5)
C12—C13—C14—C15	168.9 (3)	C64—C63—C67—O51	−165.1 (3)
C17—C13—C14—C15	47.1 (4)	C62—C63—C67—C66	−160.6 (4)
C18—C13—C14—C15	−68.2 (4)	C68—C63—C67—C66	72.8 (4)
C8—C14—C15—C16	−159.1 (3)	C64—C63—C67—C66	−45.2 (4)
C13—C14—C15—C16	−31.1 (4)	C65—C66—C67—O51	147.5 (3)
C14—C15—C16—C17	1.9 (4)	C65—C66—C67—C63	26.2 (4)
C15—C16—C17—O1	149.6 (3)	O2—C19—C69—O52	−59.3 (6)
C15—C16—C17—C13	28.2 (4)	O1—C19—C69—O52	120.6 (4)
C12—C13—C17—O1	75.2 (4)	O2—C19—C69—O51	117.4 (4)
C14—C13—C17—O1	−168.3 (3)	O1—C19—C69—O51	−62.8 (4)
C18—C13—C17—O1	−50.7 (4)	O2—C19—O1—C17	−8.1 (6)
C12—C13—C17—C16	−163.1 (3)	C69—C19—O1—C17	172.1 (3)
C14—C13—C17—C16	−46.6 (3)	C16—C17—O1—C19	135.1 (4)
C18—C13—C17—C16	71.0 (4)	C13—C17—O1—C19	−107.1 (4)
C60—C51—C52—C53	−0.6 (6)	C2—C3—O3—C3A	−6.8 (6)
C51—C52—C53—C54	0.1 (6)	C4—C3—O3—C3A	173.4 (4)
C51—C52—C53—O53	−178.9 (3)	O52—C69—O51—C67	1.1 (6)
O53—C53—C54—C55	179.7 (4)	C19—C69—O51—C67	−175.3 (3)
C52—C53—C54—C55	0.6 (6)	C63—C67—O51—C69	−118.8 (4)
C53—C54—C55—C60	−0.9 (6)	C66—C67—O51—C69	124.1 (4)
C53—C54—C55—C56	176.9 (4)	C54—C53—O53—C53A	175.1 (4)
C54—C55—C56—C57	162.9 (3)	C52—C53—O53—C53A	−5.9 (6)

### Hydrogen-bond geometry (Å, º)

**Table:**

*D*—H···*A*	*D*—H	H···*A*	*D*···*A*	*D*—H···*A*
C52—H52···O2^i^	0.95	2.51	3.161 (5)	126
C53*A*—H53*B*···O53^ii^	0.98	2.51	3.378 (6)	147
C54—H54···O2^iii^	0.95	2.56	3.309 (5)	136

Symmetry codes: (i) −*x*+1, *y*+1/2, −*z*+3/2; (ii) *x*+1/2, −*y*+1/2, −*z*+1; (iii) −*x*, *y*+1/2, −*z*+3/2.
